# Sudden Vision Loss Secondary to Optic Nerve Infiltration as a Presenting Symptom of Metastatic Lung Adenocarcinoma

**DOI:** 10.1155/2022/3614225

**Published:** 2022-11-09

**Authors:** M. M. Shamim, M. Whaley, H. Rana, S. K. Jeffus, S. Bhatti, A. B. Sallam

**Affiliations:** ^1^Department of Ophthalmology, Harvey and Bernice Jones Eye Institute, University of Arkansas for Medical Sciences (UAMS) Medical Center, Little Rock, Arkansas, USA; ^2^Hospitalist, UAMS Medical Center, Little Rock, Arkansas, USA; ^3^Department of Pathology, UAMS Medical Center, Little Rock, Arkansas, USA; ^4^Department of Oncology, Winthrop P. Rockefeller Cancer Institute, UAMS Medical Center, Little Rock, Arkansas, USA

## Abstract

**Purpose:**

To report a rare case of left-sided metastatic optic nerve infiltration and right-sided choroidal mass with exudative retinal detachment caused by EGFR exon 19 deletion positive non-small-cell lung adenocarcinoma that responded to targeted therapy with osimertinib (EGFR-TKI). Our patient demonstrated an excellent response with reduced size of the metastatic choroidal mass of the right orbit and improved visual acuity, in addition to systemic disease control.

**Case:**

A 66-year-old male patient with a history of diabetes mellitus, hypertension, and tobacco use presented with sudden vision loss in the left eye secondary to optic nerve infiltration and subacute vision loss in the right eye secondary to exudative retinal detachment from a choroidal metastasis. He was found to have a right lung mass, multiple metastatic pulmonary nodules, and liver and bone metastases. Biopsy from a mediastinal lymph node confirmed the diagnosis of metastatic lung adenocarcinoma. He was found to have exon 19 deletion on next-generation sequencing. We treated him with local radiation therapy to the left eye and systemic osimertinib (EGFR-TKI).

**Conclusion:**

To our knowledge, our case is the first report of a patient who initially presented with acute vision loss and was found to have metastatic retrobulbar optic nerve infiltration in one eye and metastatic choroidal lesion with exudative retinal detachment in the fellow eye secondary to lung adenocarcinoma. Due to the rarity of this condition, literature regarding effective treatment is scarce. Our patient demonstrated significant improvement in visual acuity and resolution of exudative retinal detachment in the right eye following osimertinib treatment and radiation therapy to the left eye. Further investigation into the role of tyrosine kinase inhibitors and radiation therapy in treating intraocular metastasis involving the optic nerve is needed.

## 1. Introduction

Intraocular metastases are commonly asymptomatic and rarely the initial presentation of a primary tumor [1]. When intraocular metastases cause vision loss, it is typically progressive rather than acute [2]. Intraocular metastasis portends a poor visual prognosis, and the majority of cases do not resolve with chemotherapy [3]. Most of the ocular metastases cases in the literature involve the uvea, orbit, or central nervous system [4]. Optic nerve infiltration secondary to metastatic lung adenocarcinoma is rare. We present a case of bilateral vision loss as the presenting symptom of lung adenocarcinoma. Our patient had choroidal metastasis with an exudative retinal detachment of the macula in one eye and a metastatic infiltration of the optic nerve without other central nervous system involvement in the fellow eye.

## 2. Case Presentation

**Figure 1:**
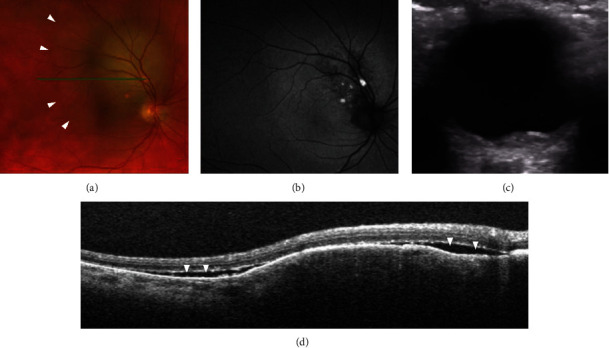
Right eye at presentation. (a) Fundus photograph showing choroidal mass and subretinal fluid (arrowheads) with (b) corresponding autofluorescence image; (c) B-scan and (d) optical coherence tomography demonstrate exudative retinal detachment (arrowheads).

A 66-year-old male with a history of diabetes mellitus, hypertension, and tobacco use presented to the emergency department with acute vision loss in the left eye and progressively decreased vision in the right eye. Visual acuity was 20/200 in the right eye and light perception (LP) in the left eye. He had a relative afferent pupillary defect (RAPD) in the left eye. Slit lamp examination of the anterior segment was unrevealing. A fundus examination showed a choroidal mass measuring approximately 6 mm × 6 mm superior to the optic nerve with an exudative retinal detachment of the right macula. Fundus examination of the left eye was unremarkable. Fundus autofluorescence revealed hyper-autofluorescent specks overlying the choroidal mass in the right eye (Figure 1). Optical coherence tomography (OCT) of the retina and B-scan ultrasonography (Figure 1) confirmed a choroidal mass and exudative retinal detachment in the right eye.

**Figure 2:**
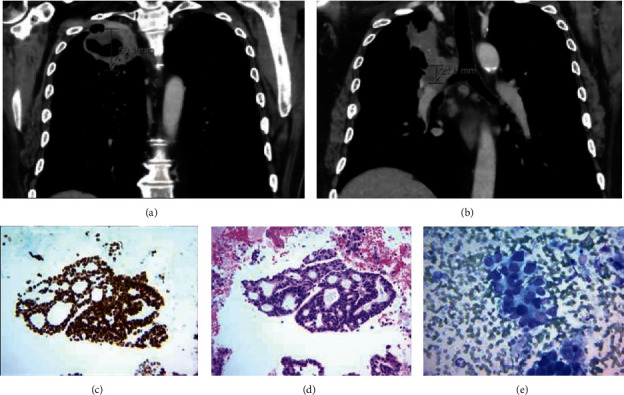
(a) Right upper lobe cavitary pulmonary lesion measuring up to 6.0 × 6.3 × 5.0 cm; (b) right superior hilar/mediastinal heterogeneously enhancing mass measuring up to 3.7 × 4.8 × 2.1 cm; (c) smear (Diff-Quik-stained slide, 400 × magnification) showing cohesive malignant cells characterized by enlarged nuclei with pleomorphism and moderate cytoplasm, consistent with metastatic non-small-cell carcinoma; (d) cell block (H&E stain, 200 × magnification) showing tumor cells that are arranged in a cribriforming growth pattern consistent with metastatic adenocarcinoma. This growth pattern has been associated with aggressive behavior; (e) immunohistochemical stain (TTF − 1, 200 × magnification) shows that the tumor cells are strongly and diffusely positive for TTF-1 (nuclear staining). This supports the diagnosis of metastasis from a lung primary (in the setting of a lung mass).

He was admitted to the hospital for further workup. CT angiogram of the head and neck revealed an incidental finding of a cavitating mass in the right upper lobe. Follow-up CT chest imaging (Figure 2) showed a large right upper lobe cavitary mass, mediastinal adenopathy, and bilateral pulmonary nodules. A diagnostic bronchoscopy was performed with a biopsy of the lung mass. The biopsy revealed lung adenocarcinoma (Figure 2).

**Figure 3:**
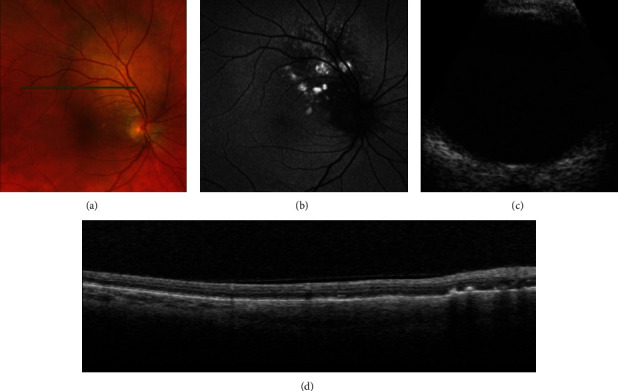
Right eye at one-month follow-up. (a) Fundus photograph showing resolution of choroidal mass with disappearance of subretinal fluid around the lesion (b) corresponding autofluorescence image; (c) B-scan showing reduction in lesion thickness and (d) optical coherence tomography demonstrating resolution of exudative retinal detachment.

MRI of the brain and orbits with and without contrast demonstrated thickening in the right posterior globe and optic nerve changes on the left side. After discharge from the hospital, the patient had PET–CT performed demonstrating PET-avid RUL lung mass with extensive pulmonary, nodal, liver, and osseous metastatic disease consistent with the clinical staging of IVB (cT3, cN2, and pM1c). A blood next-generation sequencing test was positive for an EGFR exon 19 deletion. Subsequently, he was started on osimertinib, an oral third-generation irreversible EGFR tyrosine kinase inhibitor, at a dose of 80 mg daily. The patient underwent radiation therapy to the left orbit × 3 for a total of 2400 cGy. At the patient's one-month follow-up appointment, the right eye showed complete resolution of subretinal fluid, shrinking of the right choroidal mass, and improved visual acuity in the right eye (20/30) (Figure 3).

The left eye vision did not improve, and disc pallor was noted. A posttreatment CT of the chest, abdomen, and pelvis with contrast demonstrated significant interval improvement of right upper lobe mass, improvement in mediastinal and right hilar lymphadenopathy, and improvement in metastatic liver lesions. The patient is continuing follow-up with both ophthalmology and oncology services.

## 3. Discussion

Our patient's presentation was unorthodox, with vision loss from a metastatic choroidal mass with exudative retinal detachment in the right eye and optic nerve involvement in the left. Metastases from other primary sites are the most frequent intraocular tumor in adults, with the choroid being the most common intraocular site for metastases due to its high vascular supply [5]. However, metastases to the optic nerve were shown to be extremely rare. A publication by Shields et al. with 660 cases of intraocular metastases reported a prevalence of 4.5% for optic nerve involvement [6]. Shields et al. characterized optic disc metastases as a unilateral creamy-looking infiltrate which can be associated with peripapillary hemorrhages and cotton wool spots [6]. Unique to our case is the absence of optic nerve edema in the left eye and the unremarkable appearance of the fundus despite the left RAPD indicating retrobulbar involvement of the optic nerve. Another important feature of the presented case is how the patient's subretinal fluid in the right eye resolved completely after systemic osimertinib therapy. Only the left orbit was irradiated; no irradiation was applied to the right orbit. In the FLAURA trial, osimertinib (EGFR-TKI) was shown to be beneficial for untreated, EGFR mutant advanced non-small-cell lung carcinoma, showing superior efficacy compared to earlier generation EGFR-tyrosine kinase inhibitors [7]. Osimertinib also has better CNS penetration leading to favorable CNS overall response rates and CNS progression-free survival and has previously been shown to successfully treat choroidal metastasis in a patient with EGFR-mutated lung adenocarcinoma [8].

In summary, this is the first report to our knowledge of a patient who presented with metastatic choroidal lesion with exudative retinal detachment in one eye and retrobulbar optic nerve infiltration in the fellow eye. He demonstrated significant improvement in visual acuity with resolution of exudative retinal detachment in the right eye following osimertinib therapy. Additional studies are warranted to further understand the role of tyrosine kinase inhibitors in the treatment of intraocular metastases.

## Data Availability

All data used to support the findings of this study are included within the article.
